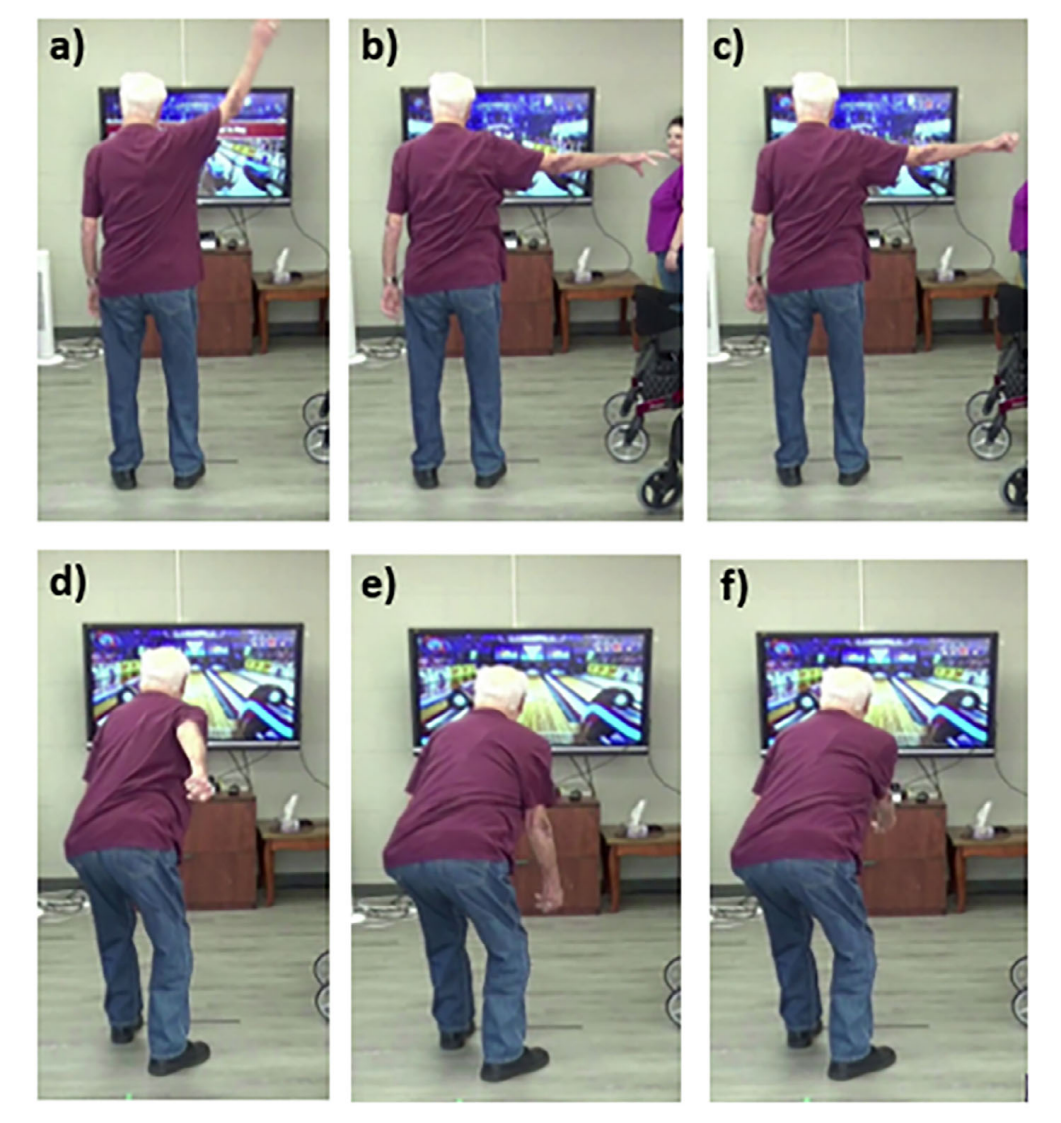# Balance or movement confidence: exergame targets for people living with dementia

**DOI:** 10.1002/alz70858_104287

**Published:** 2025-12-26

**Authors:** Erica Dove, Arlene Astell

**Affiliations:** ^1^ University of Toronto, Toronto, ON, Canada; ^2^ KITE Research Institute, Toronto Rehabilitation Institute, University Health Network, Toronto, ON, Canada; ^3^ Northumbria University, Newcastle upon Tyne, United Kingdom

## Abstract

**Background:**

Impaired balanced and cognition increase fall risk of people living with dementia [1], with experience of falls reducing confidence [2]. People living with dementia enjoy exergames, which have the potential to deliver interventions targeting balance and movement confidence [3]. This study examines the impact of a digital bowling game on balance and movement confidence of people living with dementia and explores the applicability of a novel observational approach.

**Method:**

Sixty‐six people attending four adult day programs in Ontario, Canada were recruited to a 20‐session, 10‐week digital bowling intervention (Figure 1). Pre‐ and post‐intervention, participants completed the Montreal Cognitive Assessment (MoCA), Mini Balance Evaluation Systems Test (MiniBESTest) or the Sharpened Romberg to assess balance. All sessions were video recorded. Two observational measures were developed to assess movement confidence. The first based on an existing video coding scheme was used to identify observable indicators of movement confidence (e.g., movements, tempo, and attention [4]. A second categorical measure was derived from the annotated videos, to produce a summative score out of 16 (with higher scores indicating higher confidence).

**Result:**

Participants (53% female; mean age=77.85 y; age range=58‐94 y) were living with mild to severe dementia, as indicated by their MoCA scores (mean = 12.7/30; range 0‐25), with 89% experiencing balance impairments at baseline. After the intervention MiniBESTest completers maintained their scores over time (*p* >0.05), whilst Sharpened Romberg scores declined significantly (*p* = 0.01). Movement confidence was high at the start of the intervention and remained so throughout. Participants expressed fears about falling whilst completing the balance assessments but not whilst exergaming.

**Conclusion:**

Balance and movement confidence diverged in this population, whereby participants performed better (e.g., smoother fluency of movements) in situations where their confidence was higher (i.e., exergaming). The two observational movement confidence measures appear feasible for assessing movement confidence of people living with dementia, when self‐report tools are not suitable [5]. The findings suggest that movement confidence plus more ecological approaches to measuring balance, for example whilst playing exergames, could be developed to assess interventions targeting fall risk in people living with dementia.